# The impact of COVID-19 fear during the later stages of the pandemic on maladaptive eating, psychological distress and body weight: a global cross-sectional study

**DOI:** 10.1186/s12889-025-22444-6

**Published:** 2025-04-11

**Authors:** Meeah Willig, Tomás Cabeza de Baca, Emma J. Stinson, Andrés M. Treviño-Alvarez, Theresa Rodzevik, Susanne B. Votruba, Coley C. Lameman, Jonathan Krakoff, Marci E. Gluck

**Affiliations:** 1 Phoenix Epidemiology and Clinical Research Branch, National Institute of Diabetes and Digestive and Kidney Diseases, National Institutes of Health, Phoenix, AZ USA; 2 Department of Neurology, School of Medicine, Universidad Autonoma de Nuevo Leon, Monterrey, Mexico; 3 University of Pittsburgh School of Medicine, Pittsburgh, PA USA

**Keywords:** Stress, Disordered eating, Nighttime eating, Fear of COVID-19, Depression, Binge eating, Psychosocial impact, COVID-19, Anxiety, Weight gain

## Abstract

**Background:**

The COVID-19 pandemic significantly impacted global mental health, leading to increased levels of fear, stress, and anxiety [1]. Previous research has suggested associations between functional fear of detrimental mental health outcomes and psychological stressors which may drive maladaptive eating behaviors. This study explored the associations between COVID-19 fear during later stages of the pandemic, psychological distress (anxiety, depression, and stress), maladaptive eating behaviors (emotional, uncontrolled, binge, and nighttime eating), and self-reported body weight.

**Methods:**

This was a global cross-sectional survey conducted from February 2022 to February 2024, involving 4390 adults (70% female) from 25 countries. The survey collected information on demographics, psychosocial impact, eating behaviors, and COVID-19 related behaviors. General linear models, multinomial logistic regression modes, and structural equation modeling were used to analyze the data.

**Results:**

Higher fear of COVID-19 was significantly associated with increased emotional and uncontrolled eating, even after adjusting for psychological distress and other covariates. Specifically, each unit increase in fear of COVID-19 scores was associated with a corresponding increase in emotional eating (β = 0.018) and uncontrolled eating (β = 0.029) behaviors (*p*-values < 0.0001). Furthermore, fear of COVID-19 was linked to higher odds of engaging in binge eating (OR = 1.05, 95% CI: 1.03, 1.07, *p*-value < 0.0001) and nighttime eating behaviors (OR = 1.04, 95% CI: 1.03, 1.06, *p*-value < 0.0001) in models adjusted for covariates; however, these associations were no longer significant when psychological distress variables were included. Fear of COVID-19 was also associated with body weight (β = 0.18) and BMI (β = 0.08) even with adjustments of covariates and psychological distress variables (*p*-values < 0.01). Structural equation modeling showed that fear of COVID-19 was related to current body weight through its impact on psychological distress and maladaptive eating behaviors.

**Conclusions:**

Maladaptive eating behaviors influenced by the psychological distress experienced during the COVID-19 pandemic have persisted into the later stages of the pandemic. These results underscore an interconnectedness between functional fear and its influence on maladaptive eating behaviors and body weight. Understanding this link is crucial and has the potential to inform the development of public health policies.

**Trial registration:**

Clinical Trials.gov NCT04896060 Date of Registration: May 21, 2021.

**Supplementary Information:**

The online version contains supplementary material available at 10.1186/s12889-025-22444-6.

## Background

The COVID-19 pandemic is associated with an estimated 25 to 27% increase in the prevalence of depression and anxiety across the world [1]. During the early stages of the pandemic, Rodgers et al. proposed that fear of COVID-19 combined with negative affect (i.e., anxiety, sadness, and distress) could increase eating disorder risk and symptoms in the general population [2]. Eating disorders are a range of psychological conditions characterized by abnormal eating habits, which may include emotional, uncontrolled, binge, or nighttime eating behaviors. While these maladaptive eating behaviors are not necessarily indicative of clinical eating disorders, they share underlying mechanisms and can act as coping strategies in the face of psychological distress [3].

The psychological response to stressors, including a global pandemic, is complex. Adaptive responses to threats, such as adherence to public health recommendations like hand-washing and social distancing, are often motivated by acute stress or fear [4–6]. This response aligns with the behavioral immune system, a psychological mechanism evolved to detect and respond to pathogen threats by triggering protective behaviors [7]. However, as the immediate threat diminishes, the behavioral immune system may continue to respond to perceived dangers, leading to prolonged fear and anxiety even when the actual risk is reduced. When stress becomes chronic or fear becomes excessive, these responses can transition into maladaptive states, contributing to heightened anxiety, psychological distress, and unhealthy coping mechanisms such as disordered eating [8, 9]. This prolonged psychological impact highlights the importance of studying the longer-term effects of pandemic-related fear on mental health and coping behaviors.

Individuals with heightened anxiety sensitivity—a fear of anxiety related sensations—are particularly susceptible to maladaptive coping behaviors including emotional and uncontrolled eating. Similarly, disgust responses (closely related to fear) are also higher in individuals experiencing eating disorder symptoms and are linked to anxiety sensitivity [10, 11]. This connection is particularly relevant in the context of the pandemic, as heightened anxiety sensitivity can exacerbate fear responses and contribute to maladaptive coping mechanisms like emotional or uncontrolled eating [10, 11].

Pandemics by their very nature are prolonged stressors that disrupt social norms, creating cascading effects on physical and mental health long after the immediate threat has subsided. For instance, a survey conducted between 2021 and 2022 found that survivors of the 2013–2016 West African Ebola virus epidemic had three times higher rates of mental health disorders compared to the general population of Sierra Leone [12]. During the COVID-19 pandemic, maladaptive eating behaviors, such as emotional, uncontrolled, binge, and nighttime eating, may have been used as coping mechanisms in response to pandemic-related stress and fear. Specifically, each of those eating behaviors have independently been associated with the fear of COVID-19 [13–16].

Moreover, chronic and/or excessive fear may be more harmful than short-term, momentary fear (e.g., fear experienced at the start and height of the pandemic and/or during lockdown). Communities that have experienced past epidemics and pandemics have seen persistent increases in stress, fear, and anxiety [17]. While there are an abundant number of publications demonstrating associations between COVID lockdown and increases in maladaptive eating behaviors [18], examination of the longer-term pandemic-related fear that has persisted over time remains under-explored.

This study aimed to investigate the association between fear of COVID-19 during the later stages of the pandemic and psychosocial distress (i.e., anxiety, depression, stress), weight, and maladaptive eating behaviors (i.e., emotional, uncontrolled, nighttime, and binge eating). The later stages of the pandemic, as referenced throughout this manuscript, pertain to the period during survey completion (2022–2024). During this time, vaccines were widely available, mask mandates were lifted, and COVID-19 infections were considered milder compared to those in 2020 and 2021 [19]. Unlike the aforementioned studies focused on the lockdown period, this study addresses the gap in understanding the longer-term mental health consequences of the pandemic and aims to inform strategies for mitigating these effects.

Building on previous research identifying a link between the COVID lockdown period and increased maladaptive eating behaviors, we hypothesized that fear of COVID-19 during the later stages of the pandemic would be associated with maladaptive eating disorders, including emotional, uncontrolled, nighttime, and binge eating. Furthermore, we hypothesized that fear of COVID-19 and body weight would be mediated by psychological distress and maladaptive eating behaviors, as these patterns were observed in earlier stages of the pandemic.

## Methods

### Study design

**Fig. 1:**
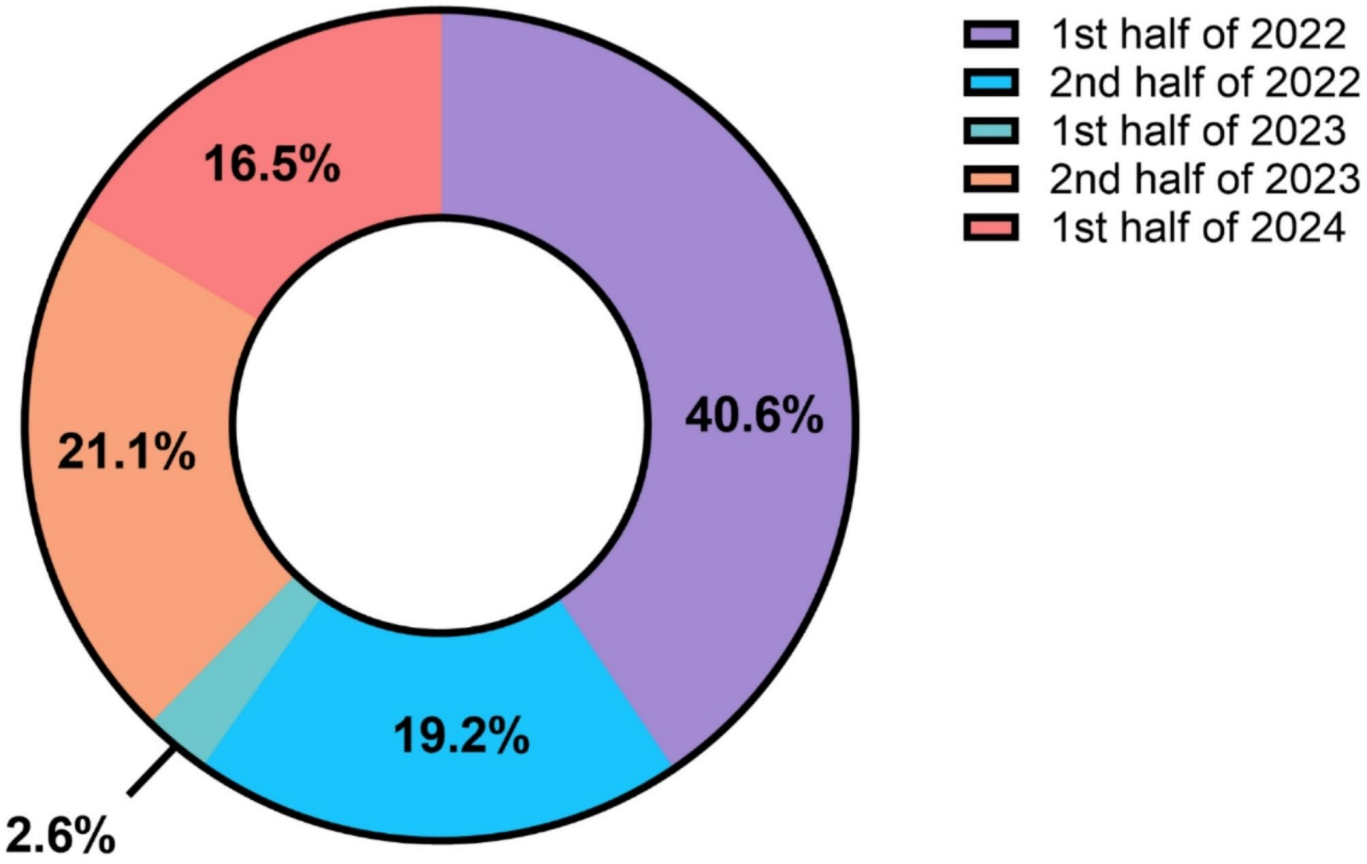
Survey completion by 6-month intervals. 1st half’ refers to January 1 to June 30, and ‘2nd half’ refers to July 1 to December 31, for the years 2022, 2023, and 2024, as shown in the legend

From February 2022 to February 2024, English-speaking adults (18 years and older) were recruited to participate in an online survey. Recruitment efforts included advertisements placed on social media accounts associated with NIDDK (e.g., NIDDK-Phoenix and NIH general accounts), as well as their listserv and ResearchMatch secure online platform. The survey assessed participants’ responses to questions about their socioeconomic standing, mental and physical health, and eating habits during the later stages of the COVID-19 pandemic (ClincalTrials.gov identifier: NCT04896060). Participation was voluntary and participants were not compensated for their participation. A total of 5017 surveys were completed. Of these, 86 were removed due to participants taking the survey twice, and one did not complete the consent correctly. This resulted in a sample of 4930 participants from 25 countries. Of those, 60% completed the survey in 2022, 24% in 2023, and 16% in 2024, (see Fig. 1). A separate cohort of participants who had previously completed a clinical research study at our institution (*n* = 52) were invited to come to our clinical research unit at the National Institute of Diabetes and Digestive and Kidney Diseases (NIDDK), Phoenix, AZ in person to fill out the survey and have their height and weight measured. All volunteers provided written informed consent before beginning the survey. This study was approved by the Institutional Review Board of the NIDDK.

The present analysis includes the online-only (*n* = 4930) due to the in-person cohort taking the survey under different circumstances (*n* = 52). Table 1. presents the descriptive statistics for the online-only cohort.

**Table 1:** Means and frequencies of covariates, predictors, and outcomes

		Race				Sex			
		Total Sample	White	Non-white	Missing	Female	Male	Other	Missing
		*N* = 4930	*N* = 3672 (75%)	*N* = 660 (13%)	*N* = 598 (12%)	*N* = 3451 (70%)	*N* = 821 (17%)	*N* = 43 (1%)	*N* = 615 (12%)
**Covariates**									
Age in years, M, (SD)		41.2 (17.1)	48.4 (17.2)***	41.1 (15.1)	44.5 (16.6)	46.2 (16.4)***	52.2 (18.6)	34.7 (12.2)	46.4 (18.2)
BMI, Median, [IQR]		27.0 [23–33]	27.0 [23–32]**	28.0 [23–35]	26.5 [24–37]	27.0 [23–33]	27.0 [24–31)	27.0 [23–35]	25.5 [23–29]
Sex, N (%)									
Female		3451 (70%)	2951 (80%)***	487 (74%)	13 (2%)	-	-	-	-
Male		821 (17%)	663 (18)	153 (23%)	5 (1%)	-	-	-	-
Other		43 (1%)	32 (1%)	9 (1%)	2 (1%)	-	-	-	-
Sex missing		615 (12%)	26 (1%)	11 (2%)	578 (96%)	-	-	-	-
Education, N (%)									
≥ High school		238 (5%)	174 (5%)	62 (9%)	2 (1%)	176 (5%)	58 (7%)	2 (5%)	2 (1%)
Some college		992 (20%)	824 (22%)	164 (25%)	5 (1%)	806 (23%)	168 (20%)	10 (23%)	8 (1%)
College+		3097 (63%)	2655 (72%)***	431 (65%)	11 (2%)	2455 (71%)***	587 (72%)	30 (70%)	25 (4%)
Education missing		603 (12%)	19 (1%)	4 (1%)	580 (96%)	14 (1%)	8 (1%)	1 (2%)	580 (94%)
Income, N (%)									
≥ $75,000		2084 (42%)	1835 (50%)***	241 (37%)	8 (1%)	1638 (47%)***	416 (51%)	13 (30%)	17 (3%)
< $75,000		2008 (41%)	1622 (44%)	375 (57%)	11 (2%)	1605 (47%)	358 (43%)	29 (68%)	16 (2%)
Prefer not to answer	212 (4%)	175 (5%)	36 (5%)	1 (1%)	174 (5%)	34 (4%)	1 (2%)	3 (1%)	
Income missing		626 (13%)	40 (1%)	8 (1%)	578 (96%)	34 (1%)	13 (2%)	0	579 (94%)
**Predictors**									
FCV-19 S, M, (SD)		14.6 (5.7)	14.3 (5.5)***	16.2 (6.5)	14.6 (6.3)	14.8 (5.6)***	13.5 (5.9)	16.8 (6.6)	14.6 (6.1)
PHQ score, M, (SD)		1.5 (1.7)	1.4 (1.7)***	1.8 (1.9)	1.2 (1.5)	1.5 (1.7)**	1.4 (1.8)	2.4 (1.9)	1.3 (1.8)
PSS score, M, (SD)		6.0 (3.7)	5.8 (3.7)***	6.7 (3.6)	6.1 (3.9)	6.1 (3.7)***	5.4 (3.7)	8.6 (3.7)	5.8 (3.3)
GAD score, M, (SD)		5.4 (5.4)	5.3 (5.3)**	6.0 (5.9)	4.1 (4.2)	5.6 (5.4)***	4.4 (5.2)	9.1 (6.4)	4.3 (5.5)
**Outcomes**									
Weight (kg), M, (SD)		79.1 (21.9)	78.7 (21.5)**	81.3 (23.9)	81.5 (21.9)	76.9 (21.7)***	88.5 (20.7)	77.9 (18.4)	71.4 (13.0)
EE score, M, (SD)		4.3 (1.3)	4.3 (1.3)	4.4 (1.3)	4.4 (1.3)	4.4 (1.3)***	4.0 (1.2)	4.3 (1.3)	4.2 (1.1)
UE score, M, (SD)		11.9 (2.7)	11.9 (2.7)	11.9 (2.7)	11.7 (2.7)	12.0 (2.8)***	11.4 (2.6)	12.4 (2.7)	10.8 (1.9)
BE, N (%)									
No BE		3295 (67%)	2815 (77%)***	467 (71%)	13 (2%)	2594 (75%)***	51 (6%)	5 (12%)	4 (1%)
BE		362 (7%)	313 (8%)	46 (7%)	3 (1%)	302 (9%)	648 (79%)	32 (74%)	21 (3%)
Missing BE		1273 (26%)	544 (15%)	147 (22%)	582 (97%)	555 (16%)	122 (15%)	6 (14%)	590 (96%)
NE, N (%)									
No NE		2758 (56%)	2416 (66%)***	331 (50%)	11 (2%)	2211 (64%)***	507 (62%)	22 (51%)	18 (3%)
NE		852 (17%)	674 (18%)	172 (26%)	6 (1%)	651 (19%)	182 (22%)	13 (30%)	6 (1%)
Missing NE		1320 (27%)	582 (16%)	157 (24%)	581 (97%)	589 (17%)	132 (16%)	8 (19%)	591 (96%)

**p-value < 0.01; ***p-value < 0.001; BE: binge eating behaviors; NE: nighttime eating

### Outcomes

#### The three-factor eating Questionnaire-18 (TFEQ-18)

We administered two subscales of the TFEQ-18 [20], which is a shortened version of the 51-item TFEQ. The emotional eating subscale (EE; 3 items; α = 0.90) measured the inability to resist eating in response to emotional distress, while the uncontrolled eating subscale (UE; 9 items; α = 0.89) assessed the tendency to overeat due to perceived loss of control overeating behaviors. Specifically, emotional eating reflected a response to psychological cues, whereas uncontrolled eating captured impulsive behaviors triggered by loss of self-regulation. Items 1–17 were scored on a 4-point scale from 1 (definitely false) to 4 (definitely true), and item 18 was scored from 1 (no restraint in eating) to 8 (total restraint). Higher scores indicated greater maladaptive eating behaviors.

#### Questionnaire on eating and weight Patterns-5 (QEWP-5)

We modified four questions from the QEWP-5 [21] to evaluate binge eating behaviors including: “In the past month, have you ever eaten what would be considered a large amount of food over a short period of time (e.g., 2 hours)?” (yes/no), “During those episodes, did you feel a sense of loss of control over your eating?” (yes/no), “About how many times per week have you had these episodes of overeating with a sense of loss of control?” (< 1x/week; at least 1d/week; 2-4x/week; or nearly everyday), and “For how long have you been engaging in these episodes at this frequency?” (less than one month/1 to 2 months/ 3 to 6 months/ or 6 months or longer) [19]. Participants were considered to have binge eating behaviors if they endorsed both eating a large amount of food (q1) and a sense of loss of control (q2) for at least 1x/week for at least 3 months or longer.

#### Night Eating Diagnostic Questionnaire (NEDQ)

Three questions from the NEDQ were used to evaluate nighttime eating behaviors: (1) “How much food do you generally eat after your evening meal as a percentage from 0-100?” (0-100%), where 100% was intended to represent eating all of one’s daily food intake after the meal (2) “Do you awake from sleep during the night and eat food?” (yes/no), and (3) “How many nights per week?” (1 day-7days) [22]. Any participant who reported consuming “25% or more” OR woke up at night to eat for at least 1 night per week or greater was considered a nighttime eater.

#### Body weight (kg) and BMI (kg/m^2^)

Participants were asked to self-report their current height and body weight. Self-reported body weight was analyzed as a separate variable in this study and was also used to calculate body mass index (BMI: weight in kilograms (kg)/ (height in meters (m))^2^). Body weights associated with BMI’s that appeared to be physiological outliers (≤ 13 kg/m^2^) were set to missing (*n* = 9).

To justify using self-reported body weights, we analyzed data from the cohort of prior NIDDK participants. This group (*n* = 52) attended an outpatient visit where they completed the online survey (which included a question to obtain self-reported weight) and had their weight measured by a clinical staff member after completion of the online survey. These two values were highly correlated (*r* = 0.96), indicating a moderate degree of accuracy and concordance between self-reported body weights in the survey when compared with objective body weight measurements.

### Covariates

This present analysis included age, race (white or non-white), sex (female, male, and other), BMI (kg/m^2^), education “high school and below,” “some college,” and “college+”, income, and survey completion year as model covariates. Annual income was categorized as being above or below $75,000, the median annual income in the U.S. during the study period [23] as well as “prefer not to answer” being an additional group. Missingness on listed covariates was accounted for by creating a category for missing data on each listed covariate (e.g., missing race was a category for race). These variables were selected because prior research identified them as either confounders or factors influencing fear of COVID-19 scores.

### Predictors

#### Fear of COVID-19 scale (FCV-19 S)

The fear of COVID-19 scale consisted of seven items that measured the extent to which a person feared COVID-19 (α = 0.88; [24]). The scale assessed various dimensions of fear, including personal health concerns, fear of losing one’s life, and physiological anxiety responses. It was scored on a 5-point Likert scale which ranged from 1 (strongly disagree) to 5 (strongly agree). Total scores ranged from 7 to 35, with a higher score indicating greater fear of COVID-19. Examples of the items included: “I am most afraid of coronavirus-19;” “I am afraid of losing my life because of coronavirus-19;” and “My heart races or palpitates when I think about getting coronavirus-19.”

#### Perceived Stress Scale-4 (PSS-4)

The 4-item version of the Perceived Stress Scale [25] (α = 0.84; [26]) assessed perceived psychological stress. Participants rated the frequency of their experiences on a 5-point scale, from 0 (never) to 4 (very often). Total scores ranged from 0 to 16, with higher scores indicating elevated levels of stress.

#### Patient Health Questionnaire-2 (PHQ-2)

The PHQ-2 is a two-item scale that evaluated depression and anhedonia over the last 2 weeks (α = 0.90) [27]. Scores ranged from 0 to 6 with higher scores corresponding to a higher risk of depression.

#### Generalized Anxiety Disorder-7 scale (GAD-7)

GAD-7 is a brief measure of anxiety (α = 0.93) [28]. The scale has seven items, and each item was scored on a 4-point Likert scale, ranging from 0 (not at all) to 3 (often). Total scores ranged from 0 to 21 with higher scores reflecting higher anxiety.

### Statistical analysis

Statistical analyses were performed using SAS 9.4 (SAS Institute Inc., Cary, NC, USA). Continuous data were presented as mean ± standard deviation (SD), except data with skewed distribution, which were expressed as median with interquartile range (IQR). Categorical data were expressed as counts and percentages. χ^2^ tests were used to analyze differences between categorical variables. General linear models (GLMs) were used to analyze differences between white, non-white, and missing race as well as female, male, other, and missing sex.

GLMs were used to examine the association between emotional eating and uncontrolled eating with fear of COVID-19 scores, accounting for covariates and predictors. GLMs were also used to look at the association between body weight and BMI, with fear of COVID-19 scores, adjusting for covariates (excluding BMI) and psychological distress variables. Results from the GLMs were displayed as unstandardized coefficients (*B*), standard error (SE), and 95% confidence intervals (CI).

Multinomial logistic regression models were conducted to assess the association between exhibiting, not exhibiting, and missing binge eating behaviors and nighttime eating behaviors with fear of COVID-19 scores. A series of 3 models (6 total) were fit for binge eating and nighttime eating. The first models were unadjusted with fear of COVID-19 as a predictor. The second models controlled for covariates including age (years), BMI (kg/m^2^), year of survey completion, sex (reference: female), race (reference: white), income (reference: $75,000 and above), and education (reference: college+). The third models included additional psychological predictors (i.e., depression, anxiety, and stress scores) as well as the covariates listed in model 2. Results were reported as odds ratios (OR) and 95% CI. Statistical significance was denoted as *p*-value < 0.05 (two-tailed).

Based on prior research, we examined whether fear of COVID-19 was associated with current weight through its relationships with psychological distress (indicated by stress, depression, and anxiety) and maladaptive eating behaviors (indicated by uncontrolled and emotional eating) by performing latent path analysis using structural equation modeling (SEM) [13, 29]. SEM assembled latent variables based on actual values for measured data. These latent variables can then be placed in a pathway based on assumptions about causality. To estimate the model, robust maximum-likelihood estimator was used due to the large sample size (*n* = 4930). This analysis was conducted using the LAVAAN package [30] in RStudio [31]. Prior to fitting the full SEM, we conducted a confirmatory factor analysis (CFA) to ensure that the observed indicators appropriately loaded onto their respective latent constructs. The SEM was assessed by χ^2^, the Tucker-Lewis Index (TLI), the Comparative Fit Index (CFI), and Root Mean Squared Error of Approximation (RMSEA), and 90% CI. Index values of the TFI and CFI between 0.90 and 0.95 are considered adequate fit and values above 0.95 are considered good fit [32, 33]. For RMSEA, values of 0.05 or less are considered indications of good fit, values between 0.08 and 0.05 are considered an adequate fit [34]. Results were presented as standardized path coefficients (β_S_).

## Results

### Participants

**Fig. 2:**
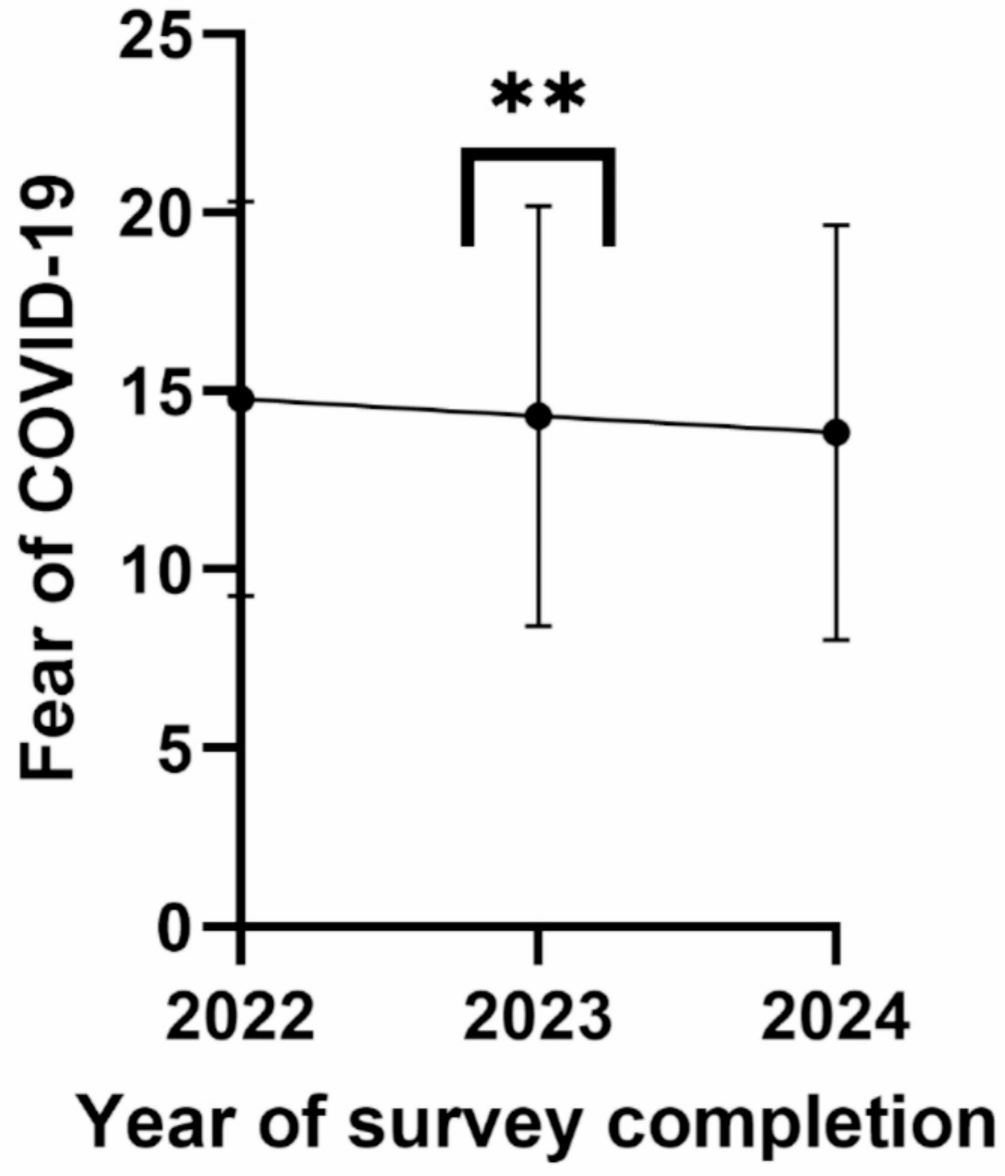
Average fear of COVID-19 scores by year of survey completion. Legend: ** *p*-value < 0.001

Participant characteristics are shown in Table 1. In brief, participants were mostly white females residing in the United States, with a mean age of 41.2 ± 17.1, a median BMI of 27 (IQR: 23–33), making over $75,000 a year, and held an undergraduate degree or higher. The mean fear of COVID-19 score was 14.6 ± 5.7 (range 7–35). Compared to participants who completed the survey in 2022, the average fear of COVID-19 score was significantly lower in those who took the survey in 2023 (*p*-value = 0.01). However, the difference between average fear of COVID-19 scores between those taking the survey in 2022 and in 2024 was not significant (*p*-value = 0.1), as illustrated in Fig. 2.

### Maladaptive eating behaviors: emotional eating and uncontrolled eating

In an unadjusted model (Model 1), a model adjusting for just covariates (age, sex, race, BMI, income, education, and survey completion year; Model 2), and a model adjusting for covariates and psychological distress variables (depression, stress, anxiety; Model 3), higher fear of COVID-19 was associated with higher emotional and uncontrolled eating scores (*p*-values < 0.0001, Table 2). When adjusting for covariates alone (Model 2), the associations became slightly attenuated, with the estimates for emotional and uncontrolled eating decreasing to β = 0.034 and β = 0.066, respectively. Further adjustment for both covariates and psychological distress variables (Model 3) led to additional reductions in the estimates for emotional and uncontrolled eating with β = 0.018 and β = 0.029, respectively.

**Table 2:** Multiple regression analyses demonstrating fear of COVID as predictor of emotional eating and uncontrolled eating

	Emotional eating score			Uncontrolled eating score		
	B	SE	95% CI	B	SE	95% CI
**Model 1**	0.042***	0.004	0.034, 0.049	0.082***	0.008	0.066, 0.098
**Model 2**	0.034***	0.004	0.026, 0.042	0.066***	0.008	0.048, 0.080
**Model 3**	0.018***	0.004	0.022, 0.055	0.029**	0.009	0.010, 0.044

**p-value < 0.01, ***p-value < 0.0001

Model 1: Fear of COVID-19 score

Model 2: Model 1 + Age + Sex + Race + BMI + Income + Survey completion year + Education

Model 3: Model 2 + Depression + Anxiety + Stress

Note: predictor variable is fear of COVID-19; outcome variables are emotional eating score and uncontrolled eating score

### Binge eating behavior and nighttime eating behavior

The multinomial logistic regression models for fear of COVID-19 and binge eating behaviors are shown in Table 3. In an unadjusted model (Model 1), higher fear of COVID-19 was significantly associated with increased odds of binge eating behavior (OR = 1.07, 95% CI: 1.05, 1.08, *p*-value < 0.0001), even after adjusting for covariates (age, sex, race, BMI, income, education, and survey completion year; OR = 1.05, 95% CI: 1.03, 1.07, *p*-value < 0.0001; Model 2). However, after adding psychological distress variables (anxiety, depression, stress) along with covariates, the association was no longer significant (OR = 1.02, 95% CI:0.98, 1.05, *p*-value = 0.08; Model 3).

As shown in Table 4, higher fear of COVID-19 was associated with increased odds of nighttime eating behavior (OR = 1.06, 95% CI: 1.05, 1.08, *p*-value < 0.0001 and OR = 1.04, 95% CI: 1.03, 1.06, *p*-value < 0.0001) in the unadjusted (Model 1) and covariate adjusted (Model 2) models, respectively, but not after adjusting for psychological distress variables (OR = 1.02, 95% CI: 1.00, 1.03, *p*-value = 0.05; Model 3).

**Table 3:** Multinomial logistic regression model, odds ratios for fear of COVID-19 as predictor of binge eating behavior

		Model 1		Model 2		Model 3	
		OR	95% CI	OR	95% CI	OR	95% CI
**Predictor: Fear of COVID-19 score**							
	Binge eating behavior	1.07***	(1.05, 1.08)	1.05***	(1.03, 1.07)	1.02	(1.00, 1.04)
	Missing	1.04***	(1.02, 1.06)	1.03**	(1.01, 1.05)	1.02	(0.98, 1.05)
	No binge eating behavior	*Ref.*		*Ref.*		*Ref.*	

**p-value  < 0.01; ***p-value  < 0.0001

Model 1: Fear of COVID-19

Model 2: Model 1 + Age + Sex + Race + BMI + income + Survey completion year + Education

Model 3: Model 2 + Depression + Anxiety + Stress

Note: predictor variable is fear of COVID-19; outcome variable is binge eating behavior

**Table 4:** Multinomial logistic regression model, odds ratios for fear of COVID-19 as predictor of nighttime eating behavior

		Model 1		Model 2		Model 3	
		OR *	95% CI	OR *	95% CI	OR *	95% CI
**Predictor: Fear of COVID-19 score**							
	Nighttime eating	1.06***	(1.05, 1.08)	1.04***	(1.03, 1.06)	1.02	(1.00, 1.03)
	Missing	1.05***	(1.03, 1.07)	1.04**	(1.02, 1.06)	1.02	(0.99, 1.05)
	No Nighttime eating	Ref.		Ref.		Ref.	

**p-value < 0.01; ***p-value < 0.0001

Model 1: Fear of COVID-19

Model 2: Model 1 + Age + Sex + Race + BMI + income + Survey completion year + Education

Model 3: Model 2 + Depression + Anxiety + Stress

Note: predictor variable is fear of COVID-19; outcome variable is nighttime eating behavior

### Body weight and BMI: GLM and structural equation modeling

**Fig. 3:**
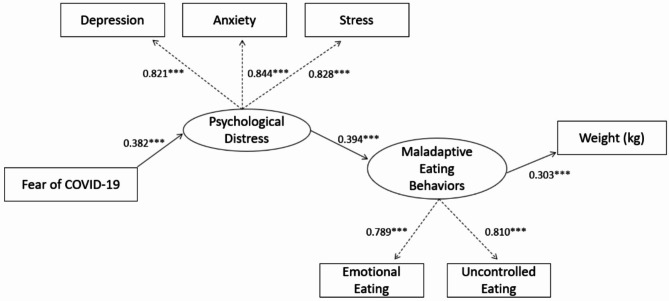
Complete path model of the association between fear of COVID-19 and weight. χ^2^ (13) = 119.155, *p* < 0.001; robust CFI = 0.985; robust TLI = 0.976; RMSEA = 0.052, 90% CI: 0.044, 0.060;*** *p*-value < 0.0001; ovals indicate latent variables and rectangles represent actual measures (i.e., indicator variables); dotted lines indicate loading onto respective latent variables; solid lines indicate standardized path coefficients (β_s_)

In unadjusted GLM models (Model 1), models adjusting for covariates (age, sex, race, income, education, and survey completion year; Model 2), and models adjusting for covariates and psychological distress variables (depression, anxiety, stress; Model 3), higher fear of COVID-19 was associated with higher body weight and BMI (all *p*-values < 0.01; Supplemental Table 1). The CFA model demonstrated excellent fit: χ^2^ (4) = 23.219, *p*-value < 0.001; robust CFI = 0.997; robust TLI = 0.993; RMSEA = 0.037, 90% CI: 0.024, 0.052. The measured values loaded significantly and highly on the designated latent variables (λ > 0.70) indicating adequate measurement of psychological distress (depression, anxiety, stress) and maladaptive eating behaviors (emotional and uncontrolled eating). The SEM model demonstrated satisfactory fit: χ^2^ (13) = 119.155, *p*-value < 0.001; robust CFI = 0.985; robust TLI = 0.976; RMSEA = 0.052, 90% CI: 0.044, 0.060. From the SEM, we assembled the casual pathway that fear of COVID-19 predicted psychological distress (β = 0.382, *p*-value < 0.001) which then predicted maladaptive eating disorders (β = 0.394, *p*-value < 0.001) which finally led to association with higher body weight (β = 0.303, *p*-value < 0.001) (Fig. 3).

## Discussion

This study investigated the longer-term effects of fear of COVID-19 on various maladaptive eating behaviors and psychological distress in a large population of individuals. Our results indicated that higher fear of COVID-19 was significantly associated with increased emotional and uncontrolled eating behaviors even after adjusting for a range of covariates and psychological distress variables. Furthermore, fear of COVID-19 was linked to higher odds of binge eating and nighttime eating behaviors, though these associations were no longer significant when accounting for psychological distress. Fear of COVID-19 was positively associated with current body weight through psychological distress and maladaptive eating behaviors (emotional and uncontrolled eating).

Our findings align with previous studies that have demonstrated an association between fear of COVID-19 and increased psychological distress, such as depression and anxiety [13, 35, 36]. However, our study extends this knowledge, by exploring the longer-term impacts of fear of COVID-19 on maladaptive eating behaviors and weight occurring in the aftermath of the lockdown. These associations between fear of COVID-19 and maladaptive eating behaviors, in particular the mediation by psychological distress variables, align with the theoretical framework proposed by Baumister et al., suggesting that fear can lead to somatization processes where psychological distress manifests as physical symptoms and behaviors (i.e., maladaptive eating behaviors) [37].Furthermore, chronic stress, such as the persistent fear of COVID-19, can lead to physiological changes that impact behavior. For instance, the physiological interactions between stress and obesity involve the stress hormone cortisol, which is produced in excess during stress and can lead to increased abdominal fat accumulation, affecting metabolic health [37]. Chronic stress can also undermine self-control and promote emotional eating. Additionally, stress can affect the reward system, particularly the mesolimbic dopamine system, which influences eating behavior and may induce a preference for high calorie foods [38]. This highlights the importance of understanding the longer-term impact of COVID-19 fear and implementing public health strategies that address both psychological and physiological aspects of stress to mitigate its adverse effects. A potential reason for the persistence of fear in the later stages of the pandemic may be attributed to several factors, including the ongoing emergence of new variants and uncertainty about their severity and transmissibility; the pandemic itself serving as a collective traumatic event, potentially leaving psychological scars; and persistent media coverage and public health messaging that may have reinforced fear even as actual risks diminished.

Our findings suggest that individuals experiencing higher levels of fear may turn to maladaptive eating behaviors as coping mechanisms to manage their psychological distress. Our SEM also supports this theory since we found that fear of COVID-19 predicted higher psychological distress which in turn predicted high engagement in maladaptive eating behaviors which then predicted higher body weight. Furthermore, our findings are substantiated by previous research indicating that emotional and uncontrolled eating can be used as strategies to cope with negative emotions and stress [39].

It is also important to note the potential bidirectionality of the associations presented in the SEM. While our model assumes that fear of COVID-19 leads to stress, which then leads to maladaptive eating and subsequent weight gain, it is also plausible that higher body weight could increase fear of COVID-19 given the heightened risk of severe disease in individuals with higher BMIs, a risk that became more apparent by 2022. Additionally, other mechanisms may have contributed to this relationship during the pandemic. For instance, individuals experiencing higher levels of stress may have faced busier schedules or disruptions to their usual routines, reducing opportunities for physical activity or healthy meal preparation. Stress can also limit access to healthier food options, particularly for those in lower socioeconomic brackets or residing in areas with limited resources. These factors may have compounded the observed associations and warrant further exploration.

This study contributes to the literature by addressing a critical gap in understanding the long-term impacts of the COVID-19 pandemic, particularly during the later stages when public health measures had been relaxed, vaccines were widely available, and COVID-19 infections were milder compared to earlier stages. Unlike many studies that focus on the acute effects of the lockdown period, our research captures the sustained psychological and behavioral impacts of the pandemic. Reports, such as *Stress in America 2023: A Nation Recovering from Collective Trauma*, highlight that mental health functioning has not returned to pre-pandemic norms, with significant increases in chronic illness and mental health diagnoses observed across age cohorts [40]. By focusing on this transitional phase, our study sheds light on how prolonged stress and fear may contribute to maladaptive coping behaviors and weight-related health outcomes, offering novel insights into the long-term consequences of pandemic-related psychological distress.

Our study is subject to limitations. Although our aim was to capture the psychosocial impact of COVID-19 on a global and diverse scale, there was a high proportion of older, highly educated, white women in our survey population. However, this demographic is particularly relevant, as there is limited information regarding disordered eating behaviors in older women. One study that examined this group found a tendency to use food as a coping mechanism in association with depressive symptoms leading to poor weight outcomes [41].

In terms of generalizability, white participants (80%) had significantly lower fear of COVID-19 scores than non-white participants (14.3 vs. 16.2, *p*-value < 0.0001). Despite this, we still found significant associations between fear of COVID-19 and maladaptive eating behaviors and self-reported body weight. Therefore, we contend that these associations may be greater in magnitude among a younger and more racially and ethnically diverse population.

Additionally, the timeframe represented in our study may reflect a unique period in the later stages of the pandemic, particularly given the smaller number of participants recruited during the first half of 2023. While the inclusion of the year of participation as a covariate in our GLM models accounted for potential variability by year, this underrepresentation may limit the interpretation of trends over time. Further investigations with a more balanced sample across years could provide additional insights into how psychological distress and maladaptive eating behaviors evolved during that period.

Also, a cross-sectional survey such as this one is only capable of creating a snapshot of the relevant data; therefore, any results cannot be considered casual in nature. Because the findings rely on self-reported answers to survey questions, there is a possibility of misinterpretation or confusion about how to respond, which should be considered when interpreting the results. Lastly, this survey collected information from people living in various states and countries, and geographical variation in infection rates, restrictions, and local pandemic conditions was not explicitly accounted for. This may limit the generalizability of our findings, as these regional differences could have influenced the levels of fear, maladaptive eating behaviors, and psychological distress reported. This study also has several strengths. In comparison to the majority of published studies reporting on behaviors observed during the lockdown period, we captured useful descriptive data regarding the later stages of the pandemic (i.e., early 2022 and after). While our sample was largely from the US, participant data was collected from all 50 states. Furthermore, we have follow-up surveys we plan to use in future investigations to examine longitudinal associations between psychological distress, maladaptive eating behaviors and weight change.

## Conclusions

Our study demonstrates that fear of COVID-19 is significantly associated with maladaptive eating behaviors, such as emotional, uncontrolled, binge, and nighttime eating. Specifically, while fear of COVID-19 independently predicted higher levels of emotional and uncontrolled eating, its association with binge and nighttime eating behaviors was largely explained by the presence of psychological distress. Moreover, fear of COVID-19 was associated with higher current weight through psychological distress and maladaptive eating behaviors. These results underscore the interconnectedness between functional fear and how it may become detrimental to mental health and influence maladaptive eating behaviors and body weight, highlighting far-reaching implications beyond the initial pandemic. These results stress the critical importance of developing effective interventions aimed at mitigating the longer-term impact of COVID-19 on individual health. Maladaptive eating behaviors can lead to a range of adverse health outcomes such as poor nutritional status, increased risk of chronic diseases, and worsened psychological well-being [42]. Implementing psychological counseling interventions can help individuals develop healthier coping mechanisms, thereby reducing the incentive of maladaptive eating behaviors.

Public health strategies should prioritize such interventions. Given that we observed strong associations in a predominantly white, highly educated study population, the effect of fear of COVID may be magnified in marginalized and disadvantaged populations. Special attention should be given to minority populations, who may be already disproportionally affected by these issues via the ongoing chronic stress from discrimination (i.e., everyday discrimination) which in itself has been shown to be associated with depressive symptomatology, maladaptive eating behaviors such as binge eating, emotional eating, and eating foods high in fat and sugar as a means of coping with this stress [43].

By addressing the functional fear as well as psychological factors underlying maladaptive eating behaviors, public health initiatives can play a crucial role in improving overall population health and resilience in the face of ongoing and future public health challenges.

## Electronic supplementary material

Below is the link to the electronic supplementary material.

Supplemental Table 1. Multiple regression analyses demonstrating fear of COVID as predictor of body weight and BMI

## Data Availability

Data described in the article will be made available upon request pending application and approval by the Institutional Review Board of the NIDDK.

## Abbreviations

NIDDK: National Institute of Diabetes and Digestive and Kidney Diseases
TFEQ-18: The Three-Factor Eating Questionnaire-18
QEWP-5: Questionnaire on Eating and Weight Patterns-5
NEDQ: Night Eating Diagnostic Questionnaire
BMI: Body mass index
kg: Kilograms
m: Meters
FCV-19S: Fear of COVID-19 Scale
PSS-4: Perceived Stress Scale-4
PHQ-2: Patient Health Questionnaire-2
GAD-7: Generalized Anxiety Disorder-7 Scale
SD: Standard deviation
IQR: Interquartile range
GLM: General linear model
β: Unstandardized coefficients
SE: Standard error
CI: Confidence intervals
OR: Odds ratio
SEM: Structural equation modelling
CFA: Confirmatory Factor Analysis
TLI: Tucker-Lewis Index
CFI: Comparative Fit Index
RMSEA: Root Mean Squared Error of Approximation
β_S_: Standardized path coefficients
