# Supplementary material for: The impact of COVID-19 fear during the later stages of the pandemic on maladaptive eating, psychological distress and body weight: a global cross-sectional study
Source: BMC Public Health. 2025 Apr 11;25:1365. doi: 10.1186/s12889-025-22444-6 (PMC11987404; doi:10.1186/s12889-025-22444-6)
Supplement: Supplementary file 1 — Supplemental Table 1. Multiple regression analyses demonstrating fear of COVID as predictor of body weight and BMI [file 12889_2025_22444_MOESM1_ESM.docx]

|  | **Body weight** | | | **BMI** | | |
| --- | --- | --- | --- | --- | --- | --- |
|  | β | SE | 95% CI | β | SE | 95% CI |
| **Model 1** | 0.22** | 0.062 | 0.10, 0.34 | 0.14*** | 0.022 | 0.10, 0.19 |
| **Model 2** | 0.26*** | 0.062 | 0.14, 0.39 | 0.11*** | 0.022 | 0.07, 0.16 |
| **Model 3** | 0.18** | 0.069 | 0.05, 0.32 | 0.08** | 0.025 | 0.03, 0.13 |
